# Review of "Handbook of Human Factors in Medical Device Design", edited by Matthew B. Weinger, Michael E. Wiklund and Daryle J. Gardner-Bonneau, Assistant Editor Loir M. Kelly

**DOI:** 10.1186/1475-925X-10-46

**Published:** 2011-06-03

**Authors:** Jonathan A Gaev

**Affiliations:** 1 Business Line Manager, BiomedicalBenchmark™, ECRI Institute, 5200 Butler Pike, Plymouth Meeting PA 19462, USA

## Abstract

Human factors is the study of the relationship between people and devices or systems. The goal of considering human factors in the design of medical devices is to create devices that take into consideration the way people use technology and process information to create a man-machine interface that leads to the best possible performance. This text describes the significant aspects of human factors issues related to medical device design. It is well written and is useful for medical device designers and for others who use or evaluate medical equipment.

## Review

Human factors is the study of the relationship between people and devices or systems. The goal of considering human factors in the design of medical devices is to create devices that take into consideration the way people use technology and process information to create a man-machine interface that leads to the best possible performance. One of the many positive results of the application of human factors to medical device design is to improve the safety of devices and to decrease use errors. This is especially important today, as more devices are used in both the hospital and home health care environment than were used many years ago. Medical devices are used now not only by trained professionals but also by many of us who have no specific education or experience with medical technology.

Here at ECRI Institute we have evaluated medical devices for over 40 years. We are a non-profit research agency and publish our results for our members and for the public without funding from the medical device or pharmaceutical industry. When we first evaluated medical technology, many devices simply didn't work. Now we find that most devices work properly but that there are differences in human factors design that significantly affect ease of use and the ability of the operator to avoid errors. Well-executed human factors design makes device operation highly intuitive and increases device safety.

I was pleased that the goal of the Handbook of Human Factors and Medical Device Design (the Handbook) is to "promote the design of safe, effective and usable medical devices". Although the primary audience is medical device designers, other professionals who seek a better understanding of human factors or who need to assess medical device ease of use will also benefit from this reference book. All major topics that directly impact the human factors issues of medical device design are presented.

The authors emphasize the importance of developing a complete understanding of the environment in order to properly design the device. They note that "environment" includes the characteristics of the user, where the device will be used and how it interacts with other devices. Many major errors in design have resulted from overlooking a key environmental issue. For example, devices designed for hospital use frequently do not work as well in the home environment because lighting and other factors may be quite different than in the hospital environment.

Since handbooks are not expected to be read "cover to cover" their organization is especially important. Each chapter of the Handbook is intended to stand on its own and includes a detailed table of contents, a general introduction to design considerations, specific design guidelines, and references to standards and to other chapters. There are 19 Chapters, each discussing a separate topic.

The first six chapters describe general topics such as "Basic Human Abilities", "Anthropometry and Biomechanics" and "Documentation". The rest of the book addresses specific topics such as "Controls", "Software User Interfaces", and "Hand Tool Design". There is a very interesting chapter entitled "Cross-National and Cross-Cultural Design of Medical Devices" that considers how people from different cultures react to colors and the placement of items on a display, among other issues. Medical device safety isn't presented in a separate chapter; it is discussed throughout the book when it relates to a particular design issue. The illustrations help support the text but unfortunately, many of them are out of focus and only a few are in color.

Using a device on a computer network is a new environmental area that is not addressed in detail in this book. When a device such as a physiologic monitor is placed on the hospital's enterprise network, the interaction with the network itself and with other technologies such as the Electronic Medical Record impacts the user's experience with the device. That interaction also needs to be analyzed from the human factors viewpoint. The software that exchanges data with the device is itself considered to be a medical device according to the Food and Drug Administration (it is called a Medical Device Data System, MDDS) so a hospital may be considered a medical device manufacturer if they create their own MDDS and would want to consider the human factors aspects of MDDS design.

The Handbook is interesting, well-written, and achieves the goal of raising the reader's awareness of important design issues. I loved the book and now that I've told my colleagues they want to read it as well, so we'll buy one for our library since I'm not giving up my copy! Although it is not intended to provide specific solutions to design problems, even a reader with experience in equipment design will most definitely gain new insights into the man-machine interface of medical devices.

